# Using MALDI-TOF mass spectrometry to identify ticks collected on domestic and wild animals from the Democratic Republic of the Congo

**DOI:** 10.1007/s10493-021-00629-z

**Published:** 2021-06-19

**Authors:** Steve Ngoy, Adama Zan Diarra, Anne Laudisoit, Guy-Crispin Gembu, Erik Verheyen, Onésime Mubenga, Sylvestre Gambalemoke Mbalitini, Pascal Baelo, Maureen Laroche, Philippe Parola

**Affiliations:** 1 Department of Zoology Centre de Surveillance de la Biodiversité, University of Kisangani, P.O. Box 2012, Kisangani, Democratic Republic of the Congo; 2 Aix Marseille Univ, IRD, AP-HM, SSA, VITROME, 19-21 Boulevard Jean Moulin, 13385 Marseille Cedex 05, France; 3 IHU Méditerranée Infection, Marseille, France; 4 EcoHealth Alliance, New York, NY 10018 USA; 5 Faculty of Sciences, University of Kisangani, Kisangani, Democratic Republic of the Congo; 6 Evolutionary Ecology Group, University of Antwerp, 2020 Antwerp, Belgium; 7 OD Taxonomy and Phylogeny, Royal Belgian Institute of Natural Sciences, 1000 Brussels, Belgium; 8 Faculté de Gestion des Ressources Naturelles Renouvelables, University of Kisangani, P.O. Box 2012, Kisangani, Democratic Republic of the Congo

**Keywords:** Ticks, MALDI-TOF MS, Arthropod identification, DR Congo, Taxonomy

## Abstract

Matrix-assisted laser desorption/ionization mass spectrometry (MALDI-TOF MS) has recently emerged as an alternative to morphological and molecular tools to identify tick species. In this study, we set out to evaluate and confirm the ability of MALDI-TOF MS to identify different species of ticks collected in the Democratic Republic of the Congo and preserved in 70% ethanol. A total of 575 ticks, of which 530 were collected from domestic pigs and 45 from wild animals, were subjected to MALDI-TOF MS analysis to evaluate the intraspecies reproducibility and interspecies specificity of MS profiles obtained from the different species. Morphologically, the ticks belonged to seven different species, namely *Rhipicephalus complanatus*, *Rhipicephalus congolensis*, *Haemaphysalis muhsamae*, *Ixodes cumulatimpunctatus*, *Amblyomma exornatum*, *Amblyomma compressum* and an unidentified *Rhipicephalus* sp. A total of 535/575 (93%) of the spectra obtained were of good enough quality to be used for our analyses. Our home-made MALDI-TOF MS arthropod database was upgraded with spectra obtained from between one and five randomly selected specimens per species. For these reference specimens, molecular identification of the ticks was also made using 16S, 12S rDNA genes and the Cox1 mtDNA gene sequencing. The remaining good quality spectra were then queried against the upgraded MALDI-TOF MS database, showing that 100% were in agreement with the morphological identification, with logarithmic score values (LSVs) between 1.813 and 2.51. The consistency between our morphological, molecular and MALDI-TOF MS identification confirms the capability and precision of MALDI-TOF MS for tick identification.

**Supplementary Information:**

The online version contains supplementary material available at 10.1007/s10493-021-00629-z.

## Introduction

Ticks are obligate blood-feeding arthropods that parasitise a large number of vertebrates, including mammals, birds, reptiles and amphibians (Mediannikov and Fenollar 2014; Parola and Raoult 2001). They are distributed into three families: Argasidae (soft ticks), Ixodidae (hard ticks) and Nuttalliellidae, representing a total of at least 898 recognised species (Dantas-Torres et al. 2012). In Africa, seven genera and 16 species of ticks are of major veterinary importance (Walker 2003). As the world’s second most important vectors after mosquitoes (Ghosh and Nagar 2014), ticks are capable of transmitting a wide variety of pathogens to humans and animals (Merino et al. 2013), such as protozoa, bacteria and viruses (Aktas 2014), which cause most emerging zoonotic infectious diseases (Ghosh and Nagar 2014).

Despite the important role that ticks play in animal and human health, tick-borne pathogens infecting humans, domestic and wild animals are poorly known in the Democratic Republic of the Congo (DRC), in Central Africa. However, some agents of tick-borne diseases such as Crimean-Congo haemorrhagic fever virus (CCHFV), *Anaplasma*, *Rickettsia*, *Theileria* and *Babesia* have been reported in the DRC in humans (Grard et al. 2011; Simpson et al. 1967; Woodall et al. 1967), domestic animals (Dahmana et al. 2019; Kalume et al. 2009, 2013; Sas et al. 2017) and tick vectors (*Rhipicephalus sanguineus* s.l. and *Amblyomma compressum*) (Mediannikov et al. 2012; Sanogo et al. 2003).

Controlling ticks and tick-borne diseases through entomological surveys relies on the accurate identification of tick species and determination of their infectious status (Parola and Raoult 2001). Tick identification occurs mainly by observing morphological characteristics or by using molecular methods (Mediannikov and Fenollar 2014; Walker 2003). However, the expertise required for the morphological identification of ticks, the impossibility of distinguishing between species of the same complex, immature or damaged specimens, the outdated of identification key, as well as the cost and length of molecular biological assays (between seven and eight hours for a sample), are limiting factors for these methods (Diarra et al. 2017).

Recently, matrix-assisted laser desorption/ionization mass spectrometry (MALDI-TOF MS) has emerged as an alternative tool for the accurate and rapid identification of many arthropod species (Yssouf et al. 2016), including mosquitoes (Diarra et al. 2019; Lawrence et al. 2019; Tandina et al. 2018; Vega-Rua et al. 2018), ticks (Boucheikhchoukh et al. 2018; Boyer et al. 2019; Diarra et al. 2017; Rothen et al. 2016), fleas (Yssouf et al. 2014; Zurita et al. 2019), sand flies (Arfuso et al. 2019; Dvorak et al. 2014; Lafri et al. 2016), triatomine bugs (Laroche et al. 2018), and lice (Ouarti et al. 2020). The effectiveness and precision of MALDI-TOF MS in identifying ticks using their legs has been reported in several studies (Boucheikhchoukh et al. 2018; Boyer et al. 2019; Diarra et al. 2017; Kumsa et al. 2016; Rothen et al. 2016; Yssouf et al. 2013). Previous studies have demonstrated the ability of MALDI-TOF MS to accurately identify several tick species from western, eastern and northern Africa (Boucheikhchoukh et al. 2018; Diarra et al. 2017; Kumsa et al. 2016). The objective of this work was to confirm the ability of MALDI-TOF MS to identify seven species of ticks collected in the DRC, stored in alcohol, and absent in our home-made MALDI-TOF MS database.

## Material and methods

### Study areas

**Fig. 1:**
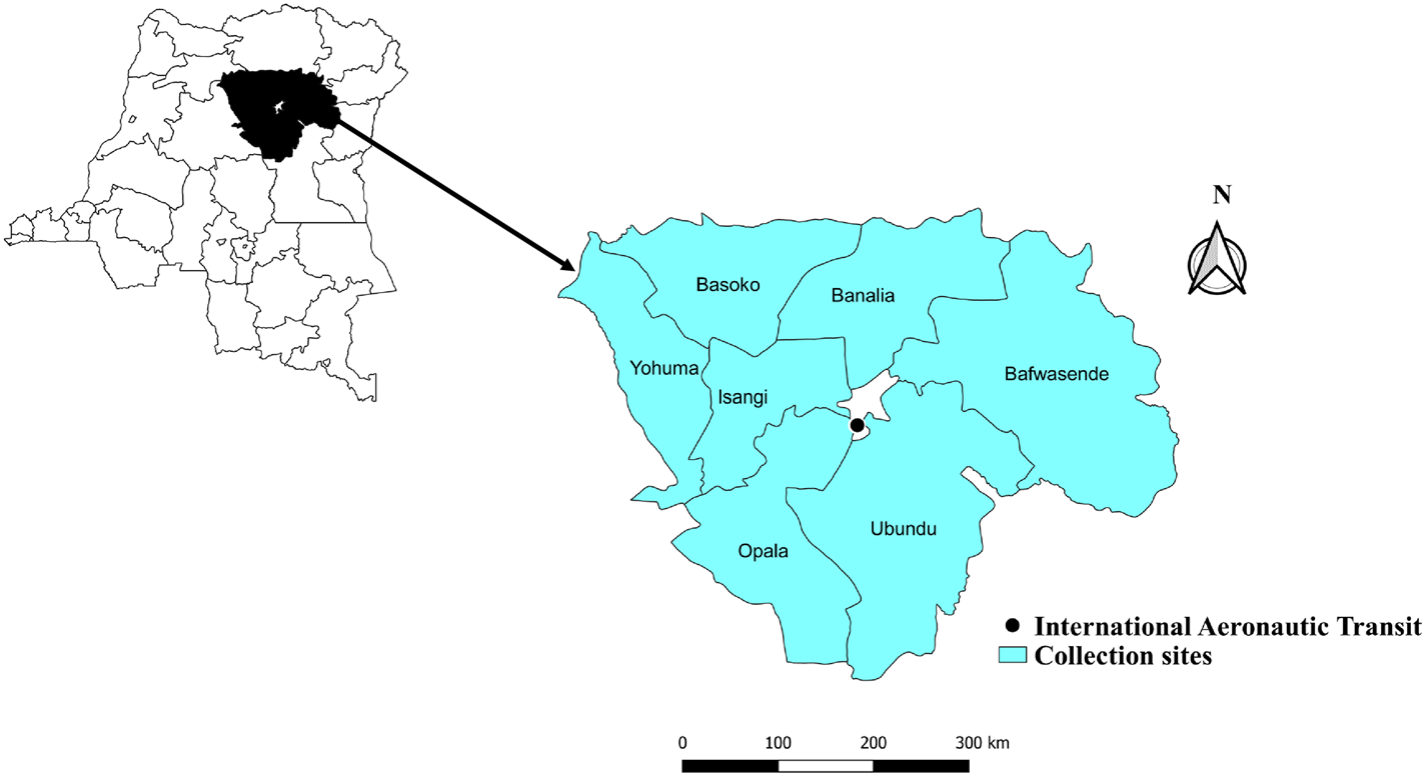
Map of the Democratic Republic of the Congo (DRC) showing the sites where the ticks used in this study were collected from domestic animals between September 2015 and September 2016 and from wild animals in 2010

The study was carried out at the slaughterhouse of the International Aeronautic Transit (IAT) market (0° 30′ N 25° 10′ E) in the city of Kisangani (0° 30′ N; 25° 11′ E) in the province of Tshopo, DRC. Tshopo is one of the 26 provinces of the DRC located in the northeast of the country and its capital city is Kisangani. About 75.5% of the population of this province lives below the poverty line. The economy is based on artisanal mining, small business and food crop agriculture, which mainly produces cassava, plantain banana, sweet potato and rice. Perennial agriculture, although in decline, is also practiced, involving coffee, cocoa, rubber and oil palm (PUND 2009). The city of Kisangani is divided into six municipalities, namely Makiso, Tshopo, Mangobo, Kabondo, Kisangani (on the right bank of the Congo River) and Lubunga (on the left bank). The IAT market slaughterhouse is in the municipality of Makiso (Fig. 1). This market is supplied by products from different cities or provinces of the DRC (Kinshasa, Mbandaka, Bumba, Basoko, etc.) via the Congo River, which plays a key role in the city’s economy. However, the domestic animals sold and butchered at the IAT market slaughterhouse mostly originate from the city of Basoko, which is surrounded by lowland tropical rainforest, and located 200 km north of Kisangani on the right bank of the Congo River (Steve et al.; unpublished).

### Tick collection and morphological identification

A total of 530 specimens of ticks were collected from 191 randomly selected domestic pigs at the IAT market slaughterhouse in Kisangani between September 2015 and September 2016. Forty-five ticks were collected from wild pangolins (*Phataginus* spp.) and monitor lizards (*Varanus niloticus*) in 2010 (Boyekoli Ebale Congo expedition collection). The ticks of wild animals protected by CITES and/or Congolese law were obtained, without financial compensation, from hunters and/or bushmeat sellers during the expedition and were covered by UNIKIS and LEGERA permits regarding the collection of biological samples. No IUCN Red List protected animals were actively trapped. Ectoparasites were removed with tweezers from fresh animal carcasses brought to one of the neighbouring bushmeat markets or encountered in the field. To collect ticks from pigs, the slaughterhouse was visited (48 visits) between 6 and 9 a.m., and visible ticks were carefully removed manually. Ticks collected from the same animal were stored in an Eppendorf vial (1.5 ml) in 70% ethanol with a host unique identifier code. In addition, data about the host such as weight, date, origin, sex, and ectoparasite type were noted in a field book and reported on digital data sheets. Approximately 20 tick specimens were sent to prof. Horak’s team at the University of Pretoria (South Africa) for morphological identification. In the DRC, the identifications made by prof. Horak’s team were extrapolated to the remaining specimens, depending on whether they were collected from the same host, in order to identify them at the species level. The ticks were transferred from IRSNB (Brussels, Belgium) to the University Hospital Institute Méditerranée infection of Marseille (Marseille, France) for analysis. In Marseille, the morphological identification of each specimen entered in the MALDI-TOF MS database was checked using a Leica EZ4 binocular microscope (Zeiss Axio Zoom.V16, Zeiss, Marly le Roi, France) and appropriate identification keys (Apanaskevich et al. 2013; Elbl and Anastos 1966a; Matthysse and Murray 1987; Walker et al. 2000). Ticks which could not be identified using morphology nor MALDI-TOF MS by the entomological team in Marseille, including two *Rhipicephalus* ticks, were sent to prof. Lorenza Beati, curator of the National Tick Collection, Institute for Coastal Plain Science, Georgia Southern University, USA, for identification.

### Sample preparation for MALDI-TOF MS and spectral analysis

After rinsing each tick specimen with distilled water and drying with sterile filter paper, all four legs on the same side of the tick were cut out with a sterile surgical blade and the tick’s body was longitudinally cut in two halves. The four legs were used for MALDI-TOF MS analysis, the half part of tick without legs was selected for molecular analysis, and the second half with the remaining legs was stored at −20 °C. Eppendorf tubes containing tick legs collected from pigs between September 2015 and September 2016 were dried at 37 °C overnight in a dry bath. The legs were then crushed using a TissueLyser device (QIAGEN, Hilden, Germany), as described previously (Nebbak et al. 2017; Diarra et al. 2017). As for ticks collected in 2010 from wild animals, the legs were crushed using the TissueLyser (QIAGEN) over six cycles of 30 ms^−1^ for 60 s in 20 μl of a mixture of 70% formic acid and 50% acetonitrile (Fluka, Buchs, Switzerland). To control matrix quality, sample loading, and performance of the MALDI-TOF mass spectrometer, the matrix solution alone was used as negative control and a protein extract from laboratory-reared *Amblyomma variegatum* legs was used as positive control. The MALDI-TOF MS spectra obtained from the tick legs were evaluated by analysing the average spectra obtained from the four spectra of each sample tested using Flex analysis 3.3, MALDI-Biotyper v.3.0. and ClinPro-tools 2.2 software. In order to verify and select MS spectra of excellent quality (i.e., peak intensity > 3000 arbitrary units, without smoothing and right baseline subtraction), the average spectra (main spectrum profile, MSP) obtained from the four spectra of each sample were visualised using flexAnalysis v.3.4 software **(**Nebbak et al. 2017). Only MS spectra with excellent quality (i.e., peak intensity > 3000 arbitrary units, without smoothing and right baseline subtraction) were selected for further analysis. The assessment of the reproducibility and specificity of the MS spectra and the ability of MALDI-TOF MS to distinguish between male and female tick specimens was performed by principal component analysis (PCA) and dendrogram analysis (cluster analysis). The PCA and dendrograms were performed using ClinPro-tools 2.2 and MALDI-Biotyper v.3.0. software, respectively, as previously described (Boyer et al. 2019).

### Database upgrading and blind tests

The spectra obtained from the legs of one to five specimens per tick species identified morphologically and molecularly were added to our laboratory’s reference spectra database, after controlling their quality, reproducibility and specificity. To create a database, MSPs reference spectra were created by combining the results of different spectra from same specimen of each species using the automated function of MALDI-Biotyper v.3.0 software (Bruker Daltonics). MSPs were created based on an unbiased algorithm using peak position, intensity and frequency data. Our entire database is currently not open access, although we are working to make it accessible to all researchers. However, it is available as part of collaborations with other researchers. In this context, the spectra files are available on request and transferable to any Bruker MALDI-TOF MS device. The reference spectra created in this study are provided in Supplementary Data 1. All good quality spectra were then blind tested against the reference spectra database. The identification level was determined using the logarithmic score values (LSVs) assigned by the MALDI-Biotyper v.3.0 software (Bruker Daltonics) (Yssouf et al. 2013). This value, ranging from 0 to 3, assesses the similarity between a tested spectrum and the reference spectra by comparing the position of the peaks and their intensity. The results of tick identification were considered reliable and relevant when the LSV was greater than or equal to 1.8 (Nebbak et al. 2017. A minimum difference of 0.2 between the best species match score and the second species match score was considered as an additional criterion for the validation of our identification (Kumsa et al. 2016).

### Molecular identification of ticks

The half-ticks were incubated individually at 56 °C overnight in 180 μl of G2 lysis buffer (Qiagen, Hilden, Germany) and 20 μl of proteinase K (Qiagen), followed by extraction using the EZ1 DNA tissue kit (Qiagen) according to the manufacturer’s recommendations. The 15 tick specimens whose spectra were entered in our database as well as 43 ticks with LSVs > 1.8 and five specimens with LSVs < 1.8 randomly selected were subjected to standard PCR, followed by sequencing using the 16s, 12s rDNA genes and the mDNA Cox1 gene (Boyer et al. 2019; Diarra et al. 2017). The forward and reverse reads of the targeted mtDNA sequences were assembled and analysed using ChromasPro v.1.34 software (Technelysium, Tewantin, Australia) and submitted for analysis to the NCBI BLAST website (http://blast.ncbi.nlm.nih.gov).

The different mtDNA gene sequences were aligned with BioEdit, and phylogenetic trees were inferred using TOPALi v.2.5 software (Biomathematics and Statistics Scotland, Edinburgh, UK) (Laroche et al. 2017b). The model Maximum Likelihood (ML) phylogenetic tree proposed default in TOPALi v.2.5 software was used for the construction of phylogenetic trees. Node numbers are percentages of the bootstrap values obtained by repeating 100 iterations of the analysis to generate a majority consensus tree (only those with a value equal to or greater than 80 were retained).

## Results

### Tick collection and morphological identification

A total of 575 ticks were collected, including 530 (92.2%) which were collected from 191 domestic pigs and 45 (7.8%) which were collected from five wild animals. Of the 530 ticks collected from domestic pigs, 452 (85.3%) were males and 78 (14.7%) were females. Of the ticks collected from wild animals, 28 (62.2%) were males, 10 (22.2%) were nymphs and seven (15.6%) were females. The morphological identification performed by the DRC team initially identified four tick species (with the limits of the method described above); namely *Rhipicephalus complanatus* (456; 79.3%), *Rhipicephalus congolensis* (61; 10.6%), *Haemaphysalis muhsamae* (one; 0.2%), and *Ixodes cumulatimpunctatus* (12; 2.1%). The two species collected from wild animals included *Amblyomma exornatum* (35; 6.1%) and *A. compressum* (10; 1.7%) (Table 1). Five specimens found on pigs were also re-examined following MALDI-TOF MS and molecular identification, and were found to belong to the *Rhipicephalus* genus, although it was not possible to identify them to the species level.

**Table 1:** Ticks collected from domestic animals between September 2015 and September 2016 and from wild animals in 2010 in the Democratic Republic of the Congo (DRC), their hosts and the result of MALDI-TOF MS identification

Tick species (Morphological ID)	Number tested (%)	Host	Number of high quality spectra (%)	Number added to database	Tick species ID by MS (number)	Log score value (average)	Mean (± SD) differences between LSV of first and second top species
*Rhipicephalus complanatus*	456 (79.3)	Pigs	444 (97.4)	5	*Rh. complanatus* (434)	1.813–2.51 (2.084)	0.66 ± 0.11
*Rhipicephalus congolensis*	61 (10.6)	Pigs	61 (100)	4	*Rh. congolensis* (57)	1.955–2.452 (2.165)	0.62 ± 0.10
*Ixodes cumulatimpunctatus*	12 (2.1)	Pigs	12 (100)	3	*I. cumulatimpunctatus* (9)	1.836–2.262 (2.011)	0.63 ± 0.18
*Haemaphysalis muhsamae*	1 (0.2)	Pigs	N/A		*Hae. muhsamae* (0)		
*Amblyomma compressum*	10 (1.7)	Pangolins	6 (60)	1	*A. compressum* (5)	1.985–2.224 (2.087)	0.85 ± 0.10
*Amblyomma exornatum*	35 (6.1)	Monitor lizards	12 (34.3)	2	*A. exornatum* (10)	1.824–2.298 (2.121)	0.86 ± 0.15
Total	575		535 (93.0)	15			

*N/A* not assigned

### MALDI-TOF MS identification

**Fig. 2:**
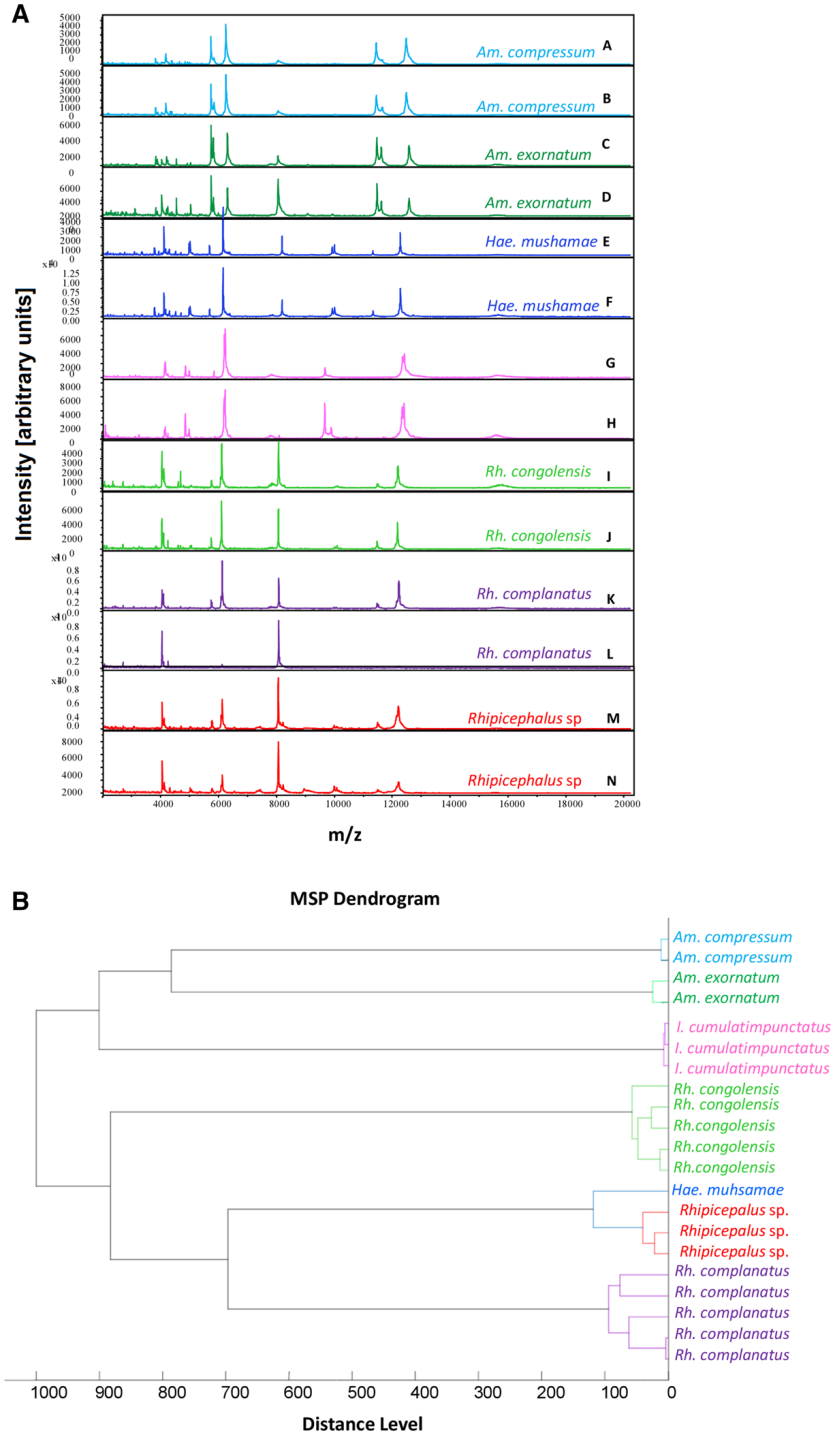
Comparison of MALDI-TOF MS spectra from different species of ticks collected from domestic animals between September 2015 and September 2016 and from wild animals in 2010 in the Democratic Republic of the Congo (DRC) using Flex analysis v.3.3 and MALDI-Biotyper v.3.0 software. **A** Representative MS spectra of *Amblyomma compressum* (A, B), *A. exornatum* (C, D), *Haemaphysalis mushamae* (E, F), *Ixodes cumulatimpunctatus* (G, H), *Rhipicephalus congolensis* (I, J), *Rhipicephalus complanatus* (K, L) and *Rhipicephalus* sp. (M, N). Abbreviations: m/z, mass-to-charge ratio; **B** MALDI-TOF MS MSP dendrogram of the seven tick species. Between one and five specimens per species were used to construct the dendrogram. The dendrogram was created using Biotyper v.3.0 software and the distance units correspond to the relative similarity of MS spectra

**Fig. 3:**
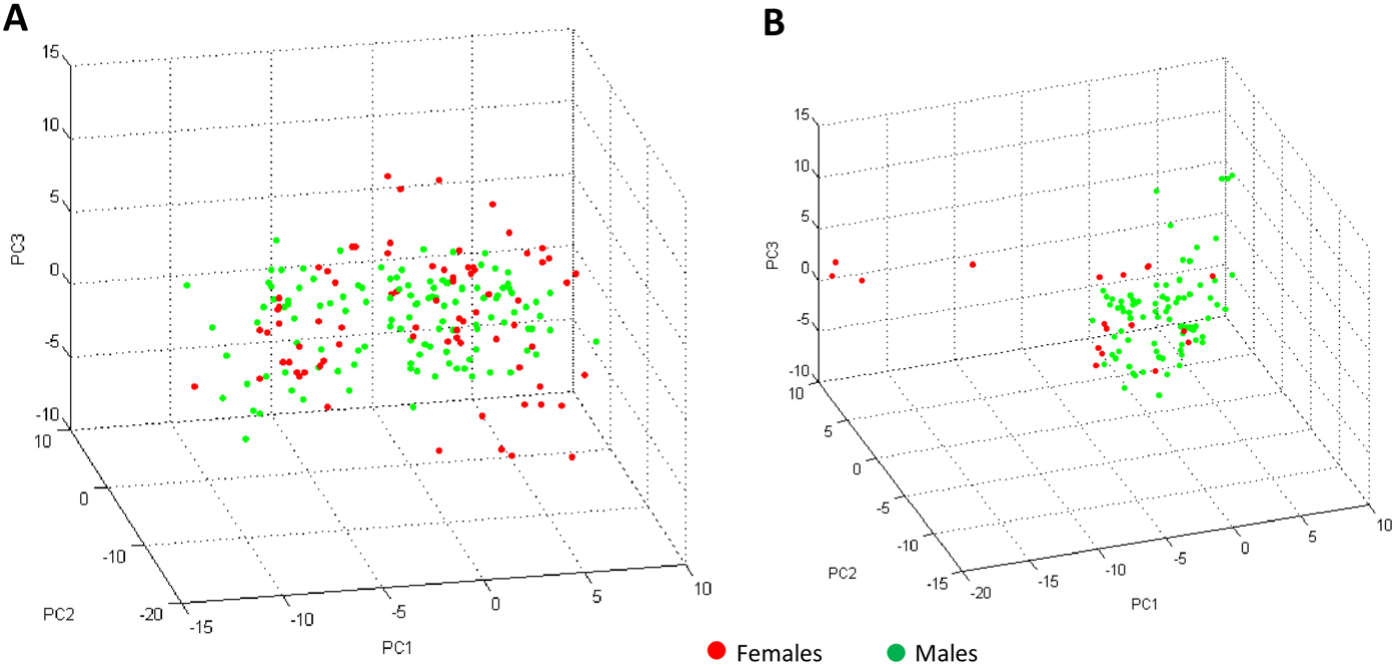
Comparison of male and female MALDI-TOF MS spectra from two different tick species collected from domestic animals between September 2015 and September 2016 in the Democratic Republic of the Congo (DRC) using the principal component analysis tool of ClinProTools v.2.2 software. **A** PCA of males (green) and females (red) of *Rhipicephalus complanatus*; **B** PCA of male (green) and females (red) of *Rhipicephalus congolensis*

All 575 ticks were subjected to MALDI-TOF MS analysis. Of these, 535/575 (93%) had good-quality spectra (Fig. 2A), including 517/530 (97.5%) ticks collected between September 2015 and September 2016, and 18/45 (40%) of those collected in 2010. Good quality spectra obtained from the legs of 15 ticks (5 *Rh. complanatus*, 4 *Rh. congolensis*, 3 *I. cumulatimpunctatus*, 2 *A. exornatum* and 1 *A. compressum*) were added to our reference database MALDI-TOF MS (Table 1). A comparative analysis of MS spectra for intra-species reproducibility and inter-species specificity was performed. The visualisation of two MS spectra per tick species showed perfect intra-species reproducibility and inter-species specificity (Fig. 2A). Similarly, the dendrogram made with the MS spectra of between one and five specimens per morphologically-identified tick species showed that specimens of the same species grouped on the same branch, confirming intra-species reproducibility and inter-species specificity (Fig. 2B). A comparison of MS spectra from male and female specimens was performed only for *Rh. complanatus* and *Rh. congolensis* species for which both sexes were available. The PCA and cluster analysis shows that there was no difference between the MS spectra of males and females (Fig. 3A, B and Supplementary Fig. 1). The remaining 520 good quality spectra of ticks were queried against our upgraded database. This blind test showed that 434/439 (98.9%) *Rh. complanatus*, 56/56 *Rh. congolensis*, 9/9 *I. cumulatimpunctatus*, 5/5 *A. compressum* and 12/12 of *exornatum* were correctly identified by MALDI-TOF MS, with LSVs between 1.813 and 2.51 (Table 1). Thus, 99% (515/520) of the ticks which were blind tested presented a concordance with the morphological identification and were considered correctly identified.

The morphology of the five ticks which were initially morphologically identified as *Rh. complanatus* that were not correctly identified by MALDI-TOF MS (LSV < 1.8) MALDI-TOF MS were rechecked, using the remaining part of the body. This revealed that one tick was in fact *Hae. muhsamae* and that four identical specimens belonged to another *Rhipicephalus* sp., which could not be definitively identified to the species level. The spectra of these five specimens did not match any of the ticks in our MALDI-TOF MS database and had LSVs ranging from 1.00 to 1.327. The spectrum of *Hae. muhsamae* was added to the MALDI-TOF MS database. The spectrum of one *Rhipicephalus* sp. was also temporarily added to the database as a potential reference spectrum for the remaining *Rhipicephalus* sp. The blind test showed that the three other unidentified *Rhipicephalus* sp. indeed matched with the spectrum of the *Rhipicehalus* sp. introduced in the database, with LSVs ranging from 2.05 to 2.22. The mean differences between the first species match scores and the second species match scores were 0.66 ± 0.11, 0.62 ± 0.10, 0.63 ± 0.18, 0.86 ± 0.15 and 0.85 ± 0.10 for *Rh. complanatus*, *congolensis*, *I. cumulatimpunctatus*, *A. exornatum* and *A. compressum*, respectively (Table 1). We had 100% agreement between our morphological identification and MALDI-TOF MS for the specimens tested.

### Molecular identification of ticks

**Fig. 4:**
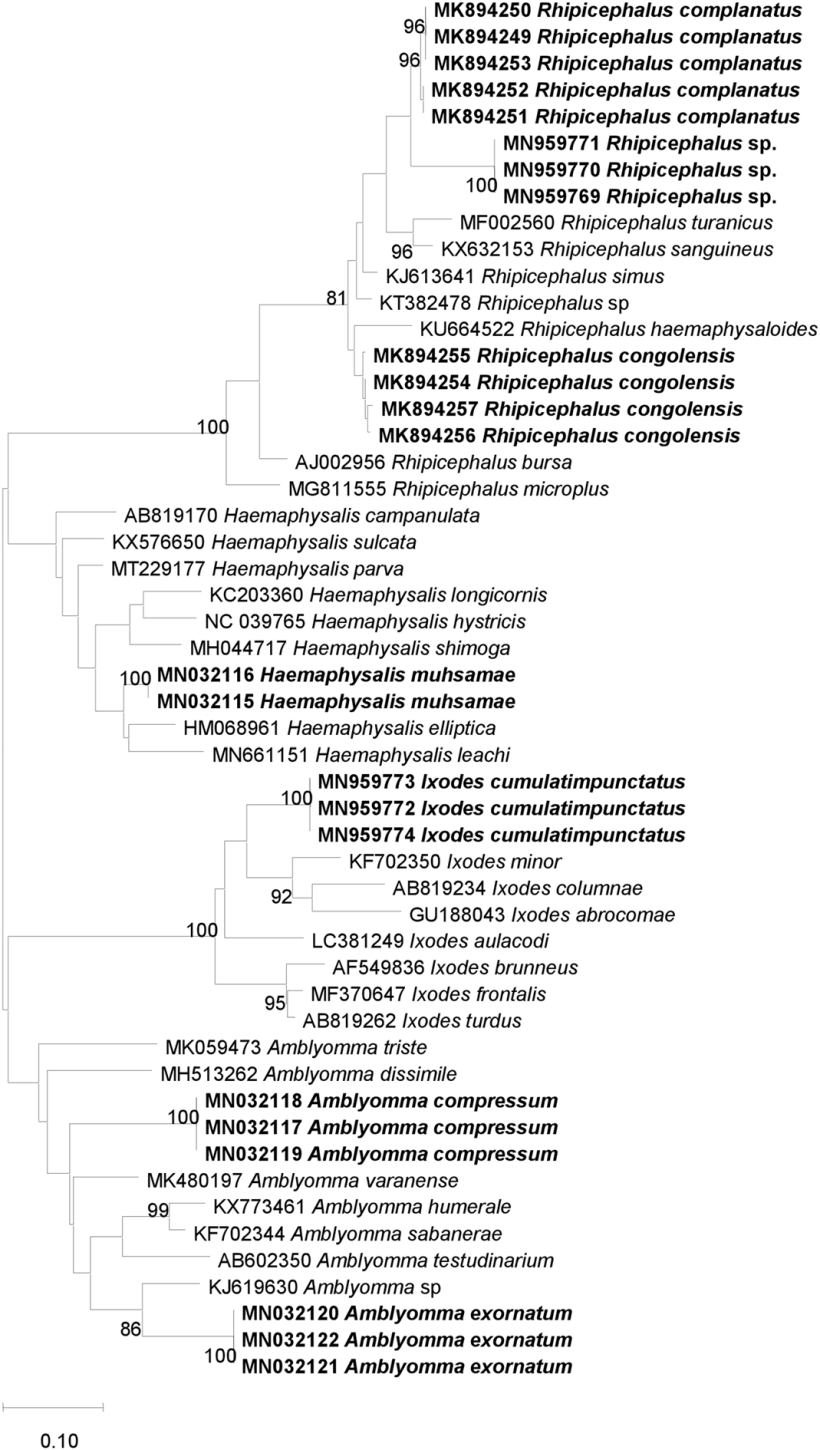
Phylogenetic analysis based on the 410 base pair (bp) fragment of the 16s ribosomal RNA gene of ticks collected from domestic animals between September 2015 and September 2016 and from wild animals in 2010 in the Democratic Republic of the Congo (DRC). The tree was generated using the Maximum Likelihood algorithm (PhyML) with the general time-reversible (GTR) model proposed by TOPALi v.2.5 software. The specimens in our study are shown in bold and are preceded by the GenBank accession numbers of each sequence

**Fig. 5:**
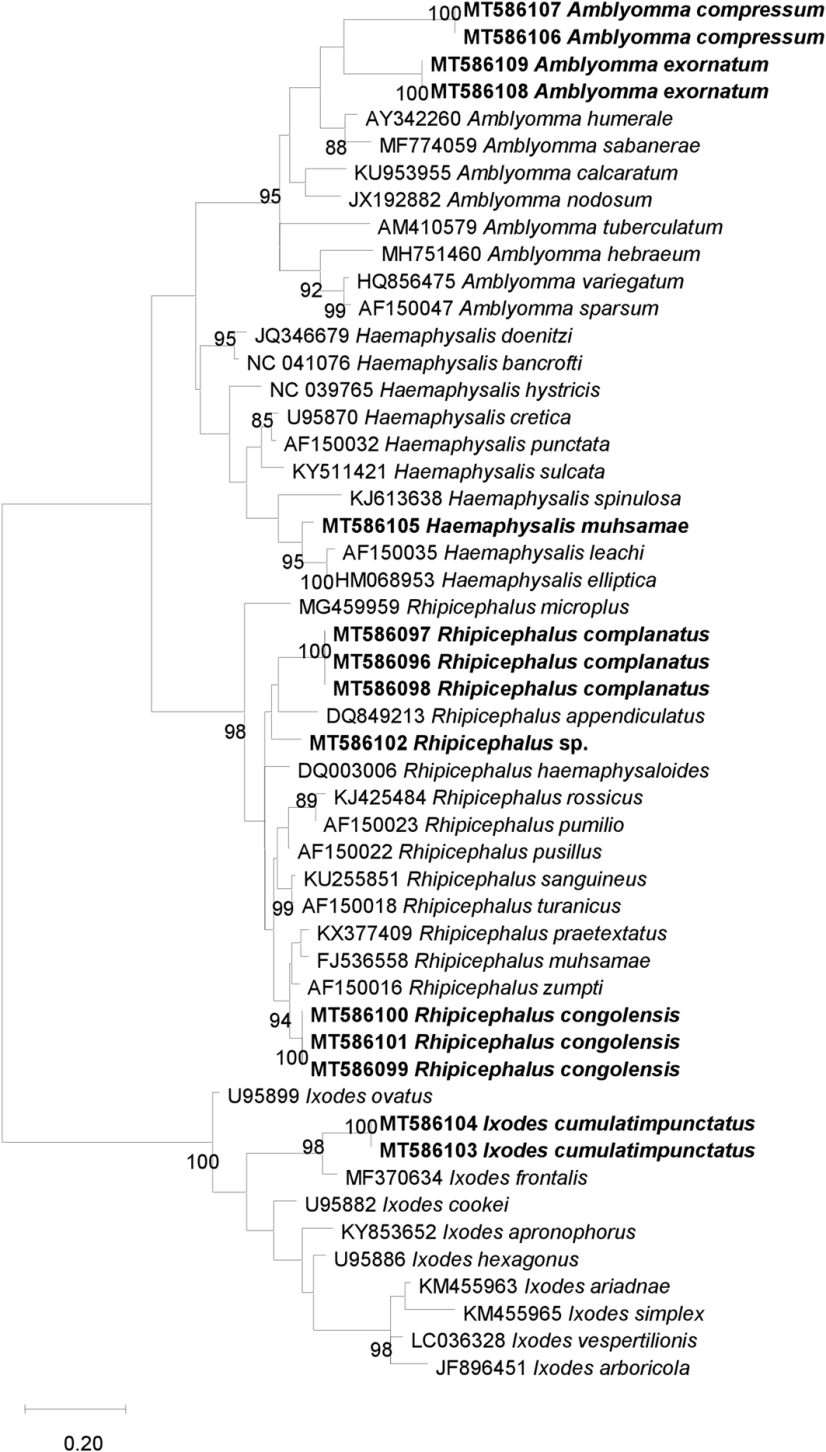
Phylogenetic analysis based on the 340 bp fragment of the 12s ribosomal RNA gene of ticks collected from domestic animals between September 2015 and September 2016 and from wild animals in 2010 in the Democratic Republic of the Congo (DRC). The tree was generated using the Maximum Likelihood algorithm (PhyML) with the general time-reversible (GTR) model proposed by TOPALi v.2.5 software. The specimens in our study are shown in bold, and are preceded by the GenBank accession numbers of each sequence

**Fig. 6:**
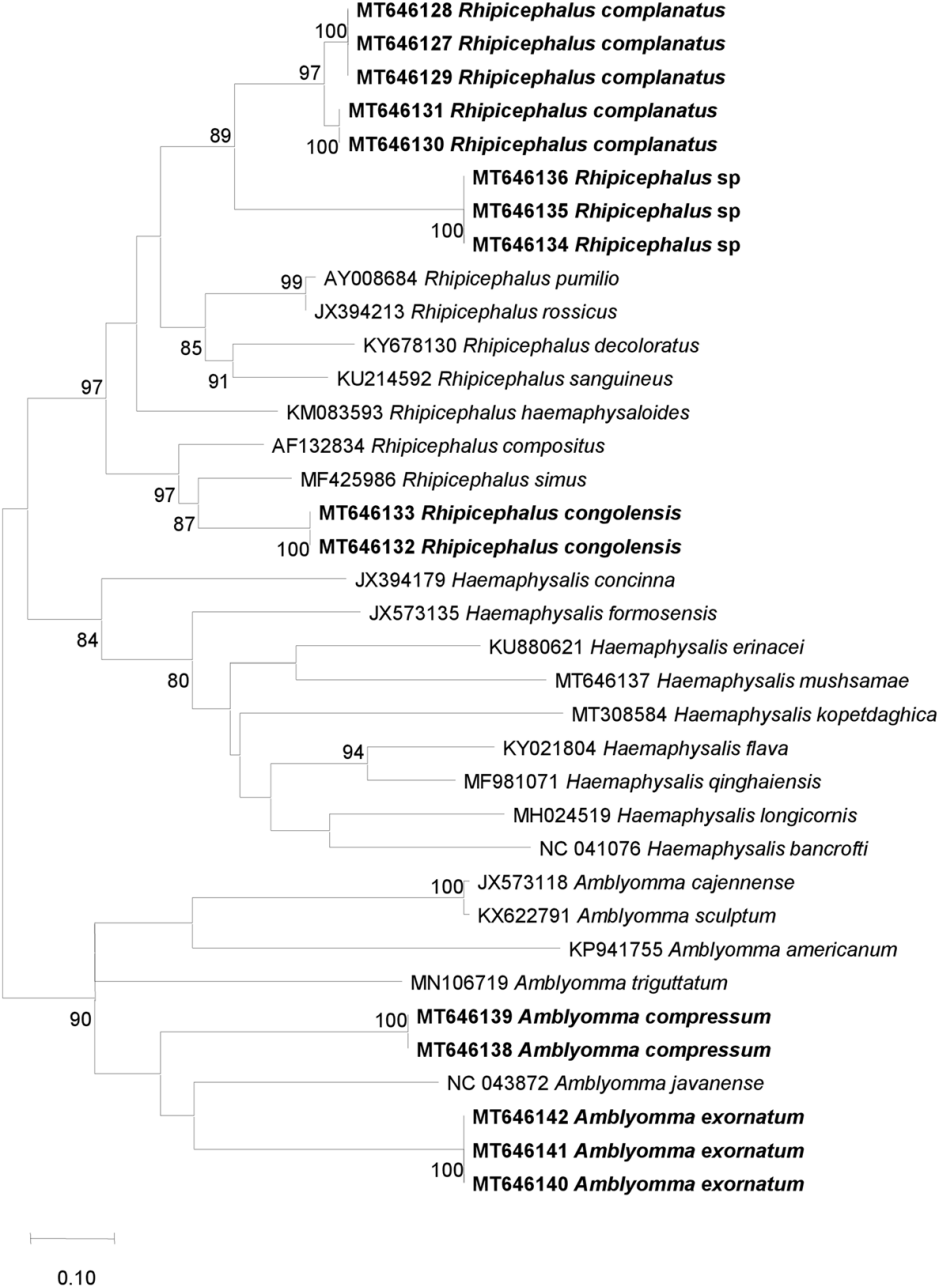
Phylogenetic analysis based on the 720 bp fragment of the Cox1 mtDNA gene of ticks collected from domestic animals between September 2015 and September 2016 and from wild animals in 2010 in the Democratic Republic of the Congo (DRC). The tree was generated using the Maximum Likelihood algorithm (PhyML) with the general time-reversible (GTR) model proposed by TOPALi v.2.5 software. The specimens in our study are shown in bold and are preceded by the GenBank accession numbers of each sequence

Fifty-seven ticks, including 25 *Rh. complanatus*, 14 *Rh. congolensis*, 5 *I. cumulatimpunctatus*, 4 *A. exornatum*, 3 *A. compressum*, 2 *Hae. muhsamae* and 4 *Rhipicephalus* sp., were subjected to standard PCR and sequenced using the 16s, 12s rDNA gene and the Cox1 mtDNA gene. We obtained sequences for all the specimens using the 16s rDNA gene, while only 15 were obtained with the 12s rDNA gene (3 *Rh. complanatus*, 3 *Rh. congolensis*, 2 *I. cumulatimpunctatus*, 2 *A. exornatum*, 2 *A. compressum*, 1 *H. muhsamae* and 1 *Rhipicephalus* sp.) (Table 2). We obtained 22 sequences when targeting the Cox1 mtDNA gene, including 9 *Rh. complanatus*, 2 *Rh. congolensis*, 4 *A. exornatum*, 2 *A. compressum*, 1 *Hae. muhsamae* and 4 *Rhipicephalus* sp. Blast results showed that the percentages of sequence identities ranged from 85.3 to 96.6%, 85.6 to 96.8% and 86.7 to 90.6%, respectively, for genes 16s, 12s rDNA and Cox1 mtDNA, with the known species available on GenBank. This DNA-based approach did not allow us to identify the *Rhipicephalus* sp. ticks that had not been morphologically identified down to the species level. The percentage of sequence identity of these ticks was 92.2, 93.5 and 87.4% with the sequences of *Rh. sanguineus* (KT382455), *Rh. pusillus* (AF150022) and *Rh. sanguineus* (KU364312), respectively, for genes 16s, 12s rDNA and Cox1 mtDNA (Table 2). These BLAST results were confirmed by building phylogenetic trees, and these trees were consistent with the BLAST results (Figs. 4, 5 and 6). The sequences of the three genes of each tick species, excluding *I. cumulatimpunctatus*, were deposited in GenBank and the accession numbers are available in Supplementary Table 1.

**Table 2:** Results of the molecular identification of tick specimens collected from domestic animals between September 2015 and September 2016 and from wild animals in 2010 in the Democratic Republic of the Congo (DRC), using the 16s, 12s rDNA and Cox1 mtDNA genes

	16s rDNA gene				12s rDNA gene				Cox1 mtDNA gene			
	Number sequenced (sequences obtained)	Similarity between sequences	Molecular identification (% identity)	Accession number	Number sequenced (sequences obtained)	Similarity between sequences	Molecular identification (% identity)	Accession number	Number sequenced (sequences obtained)	Similarity between sequences	Molecular identification (% identity)	Accession number
*Rhipicephalus complanatus*	25 (25)	99.26–100%	*Rh. sanguineus* (93.87%)	KT382455	25 (3)	100%	*Rh. turanicus* (92.08%)	AF150018	25 (9)	97.05–100%	*Rh. sanguineus* (88.76–89.31%)	KU364312/ KU214592
*Rhipicephalus congolensis*	14 (14)	99.26–99.56%	*Rh. simus* (95.80–96.56%)	KJ613641	14 (3)	99.41–100%	*Rh. simus* (96.76%)	AF150019	14 (2)	100%	*Rh. simus* (90.56%)	MF425986
*Ixodes cumulatimpunctatus*	5 (5)	100%	*I. turdus* (90.27%)	AF549838	5 (2)	100%	*I. frontalis* (90.78)	MF370634	5 (0)	/		
*Rhipicephalus sp.*	4 (4)	99.75–100%	*Rh. sanguineus* (92.20–92.44%)	KT382455	4 (1)	/	*Rh. pusillus* (93.53%)	AF150022	4 (4)	100%	*Rh. sanguineus* (87.36%)	KU364312
*Haemaphisalis muhsamae*	2 (2)	100%	*Hae. elliptica* (85.37%)	HM068961	2 (1)	/	*Hae. elliptica* (94.99%)	HM068953	2 (1)	/	*Hae. erinacei* (86.76%)	KU880621
*Amblyomma compressum*	3 (3)	100%	*A. varanense* (86.74%)	MK480197	3 (2)	100%	*A. multipunctum* (85.63%)	KM077433	3 (2)	100%	*A. trigultatum* (86.70%)	MN106719
*Amblyomma exornatum*	4 (4)	100%	*Amblyomma* sp. (87.98%)	KJ619630	4 (2)	100%	*Amblyomma* sp. (87.57%)	AY766150	4 (4)	100%	*Rh. appendiculatus* (86.66%)	KC503257

## Discussion

Recently, MALDI-TOF MS has been proposed as an accurate, rapid and economical tool for the identification of many arthropods of medical interest, including ticks (Yssouf et al. 2016). Although the acquisition of a MALDI-TOF mass spectrometer can be expensive, its use for entomological analyses involves low additional costs. The reagents used for this high-throughput technique are indeed inexpensive, and the sample preparation and the data analyses are simple and fast compared to morphological and molecular methods (Laroche et al. 2017a).

MALDI-TOF MS is a method which can generate a specific protein signature of the analysed organism. It is an approach that allows for the rapid analysis of large sets of samples of a priori unidentified arthropods and provides a specific spectrum for each arthropod species that can be compared against a library of reference spectra of reliably identified specimens (Boucheikhchoukh et al. 2018). MALDI-TOF MS has been used to identify ticks that had been frozen or preserved in alcohol, and collected from various countries (Boucheikhchoukh et al. 2018; Boyer et al. 2019; Diarra et al. 2017; Karger et al. 2012; Kumsa et al. 2016; Yssouf et al. 2013). Currently, the challenges of this technique remain selecting the body part to be used, the methods of arthropod conservation in order to generate sufficiently reproducible and specific spectra, and the need to have a database of arthropod spectra formally identified by morphology and confirmed by molecular biology, if possible (Laroche et al. 2017a).

In this study, we morphologically identified six different species, four of which were collected from domestic pigs (*Rh. complanatus*, *Rh. congolensis*, *Hae. muhsamae* and *I. cumulatimpunctatus*) and two from wild pangolins and monitor lizards (*A. exornatum* and *A. compressum*). *Rhipicephalus complanatus* is a tick that almost exclusively parasitises domestic and feral pigs. This tick is mainly found in the DRC and neighbouring countries to the west such as Cameroon, Gabon, the Central African Republic and Congo. It has also been reported in Côte d'Ivoire, Liberia, and western Uganda (Walker et al. 2000). *Rhipicephalus congolensis* is a recently described tick that parasitises red pigs and domestic pigs in the DRC (Apanaskevich et al. 2013). *Haemaphysalis muhsamae* is a parasite of birds, carnivores, insectivores, rodents and lagomorphs (Elbl and Anastos 1966b; Uilenberg et al. 2013). This tick has been reported in the Guinean and eastern savannah zones in areas with at least 1 000 mm of annual rainfall and at different altitudes (Elbl and Anastos 1966b; Uilenberg et al. 2013). *Haemaphysalis muhsamae* has not currently been reported on domestic animals, and we are reporting it for the first time on domestic pigs from the DRC. *Ixodes cumulatimpunctatus* is a non-specific polytropic tick which, in its adult stages live on mammals and in its immature stages, lives on mammals and birds. This tick is widespread throughout the west African lowland rainforest and high altitude wooded savannas from Uganda to Zimbabwe (Cumming 1999; Morel and Mouchet 1965). *Amblyomma exornatum* ticks are mainly found on reptiles, mostly monitor lizards, lizards and pythons. They can also be found on tortoises, crocodiles, chameleons, and also on mammals (bats, pangolins, rodents) and ungulates (cattle and carnivores) (Nowak-Chmura 2014). *Amblyomma exornatum* is widespread in Africa and has been reported in Algeria, Senegal, the Côte d’Ivoire, Ghana, Cameroon, Gabon, Congo, Somalia, Kenya, Angola, Tanzania, Botswana, Mozambique and the Republic of South Africa (Nowak-Chmura 2014). *Amblyomma compressum* is a tick species which has thus far exclusively been reported for pangolin species, including the ground pangolin (*Smutsia temminckii*), tree pangolin (*Manis tricuspis*) and giant pangolin (*Manis gigantea*) (Uilenberg et al. 2013). This tick has been found in Central Africa (DRC, Rwanda, Gabon and Burundi) (Elbl and Anastos 1966a; Uilenberg et al. 2013; Pourrut et al. 2011) and also in West Africa (Ghana, Cote d’Ivoire, Liberia and Nigeria) (Elbl and Anastos 1966a; Mediannikov et al. 2012; Ntiamoa-Baidu et al. 2004; Uilenberg et al. 2013).

In our study, 93% of the ticks submitted to MALDI-TOF MS analysis yielded good quality spectra. The percentage of good quality spectra depends on the storage time of the ticks in alcohol. This percentage higher (97%) for ticks from 2015 and 2016 and lower (40%) for those stored since 2010. The performance of tick identification is not particularly impacted by the alcohol 70% because with an adapted database, we obtained identification percentages very similar to those of fresh ticks, often around 100% (Boucheikhchoukh et al. 2018; Diarra et al. 2017; Kumsa et al. 2016). The impact of alcohol on MS MALDI-TOF profiles, resulting in lower intensity and overall quality than fresh or frozen samples, has been reported in previous studies (Diarra et al. 2017; Kumsa et al. 2016; Nebbak et al. 2017; Rothen et al. 2016). Despite the impact of the preservation method on the quality of the MALDI-TOF MS profiles, MALDI-TOF MS appears to be efficient, fast and less expensive for the identification of ticks if a specific database is created with good quality spectra of specimens preserved under the same conditions, as demonstrated by other studies (Diarra et al. 2017; Kumsa et al. 2016).

In this study, we observed no differences in MS spectra from male and female *Rh. complanatus* and *Rh. congolensis*. A previous study reported that it was not possible to definitively distinguish between male and female ticks on the basis of their MS spectra (Yssouf et al. 2013). Nevertheless, differentiation of male and female mosquito MS profiles has been achieved (Fall et al. 2021). More in-depth analysis of fresh tick spectra should be performed in order to assess whether MALDI-TOF MS is actually able to distinguish male and female specimens.

Based on morphological identification, all six of these tick species were not represented in our home-made reference spectra MALDI-TOF MS database. With the exception of *Hae. muhsamae,* for which we did not have good quality spectra, the reference spectra of five species, namely *Rh. complanatus*, *Rh. congolensis* and *I. cumulatimpunctatus,* collected in 2015 and 2016, and *A. exornatum* and *A. compressum* collected in 2015 were added to our MS MALDI-TOF home-made database. In the first step, 515/520 ticks were correctly identified at species level with LSVs > 1.8, which is the threshold for reliable identification of arthropods (Yssouf et al. 2013). The mean differences between the first and second species match scores in our study were > 0.2, which is the minimum difference to distinguish two species (Kumsa et al. 2016). The five unidentified specimens were due to morphological identification errors highlighted by the MALDI-TOF MS tool. MALDI-TOF MS incited us to re-examine the morphology of these ticks which were morphologically identified as *Rh. complanatus*. We were the able to identify one of the five specimens as *Hae. muhsamae*. None of the three experts in tick identification involved in this study was able to morphologically identify the remaining four specimens of *Rhipicephalus* sp. to the species level. However, it should be noted that these ticks were engorged females, whose morphological characteristics were distorted or had disappeared, making identification impossible, highlighting the limits of morphological identification (Walker et al. 2003). After temporarily adding the spectrum of one of these specimens in the database, MALDI-TOF MS confirmed that all four belonged to the same species.

Knowing that the species which were morphologically identified in our study were not yet represented in the GenBank database, we sequenced the specimens chosen for the creation of the MALDI-TOF MS database, as well as others with confirmed MALDI-TOF MS identification, by amplifying part of the 16s and 12s rDNA genes and the Cox1 mtDNA gene, as previously used to discriminate ticks (Boyer et al. 2019; Diarra et al. 2017). This allowed us, on the one hand, to evaluate the percentage of similarity of sequences from the same tick species for the three genes, and, on the other, to compare the identity of all these sequences with those available in the GenBank database to reinforce the results. The high percentage of similarity between the sequences of different specimens of the same species (97% to 100%) using the three genes supports the robustness of our morphological and MALDI-TOF MS identification. However, the low identity of our sequences (between 85 and 95%) with the reference sequences of the 16s, 12s rDNA and Cox1 mtDNA genes of the tick species available in the GenBank database could be due to the absence of sequences of the tick species from our study in this database. The phylogenetic trees constructed with the sequences of the three genes clearly show that *Rhipicephalu*s sp. form a separate group from *Rh. complanatus* (Figs. 4, 5 and 6), and thus have different genomic sequences. Similarly, the three genes used did not allow us to identify the four specimens of *Rhipicephalus* sp., which we could not identify down to the species level by either morphology or MALDI-TOF MS, thus showing the limitations of this technique, namely the insufficient number of reference sequences and the reliability of the morphological identification of tick species whose sequences already available on GenBank (Yssouf et al. 2016).

MALDI-TOF MS has been used in clinical laboratories in developed countries and capacity building was performed in Senegal, where staff and students was trained to use this technique for bacterial and entomological identifications. It can indeed be used in medical entomology, with almost zero additional costs, for the rapid and accurate identification of arthropods and determination of their trophic preferences and infection status (Yssouf et al. 2016). It is a tool that also makes it possible to correct morphological identification errors, as was the case in this study, where the four specimens of *Rhipicephalus* sp. were not recognised after the blind test, while a fine analysis of the spectral profiles of these ticks showed that they were reproducible and grouped together.

## Conclusion

The results of this study show consistency between our MALDI-TOF MS identification and the morphological and molecular identifications, confirming once again that MALDI-TOF MS is an reliable tick identification tool. Four specimens of *Rhipicephalus* sp. could not be identified by MALDI-TOF MS but neither could they be identified by morphology or molecular biology, thus demonstrating the importance and interdependance of all these approaches. However, we showed that, for ticks that have been kept in alcohol for a very long time, the MALDI-TOF MS sample preparation had to be adjusted, i.e., a reduction in the quantity of the mix and increased crushing time resulted in an improvement of the quality of the spectra.

## Supplementary Information

Below is the link to the electronic supplementary material.

Supplementary file1 (TIFF 688 KB)

Supplementary file2 (DOCX 32 KB)

Supplementary file3 (RAR 2866 KB)
