# Supplementary figures and images for: Using MALDI-TOF mass spectrometry to identify ticks collected on domestic and wild animals from the Democratic Republic of the Congo
Source: Exp Appl Acarol. 2021 Jun 19;84(3):637–57. doi: 10.1007/s10493-021-00629-z (PMC8257524; doi:10.1007/s10493-021-00629-z)

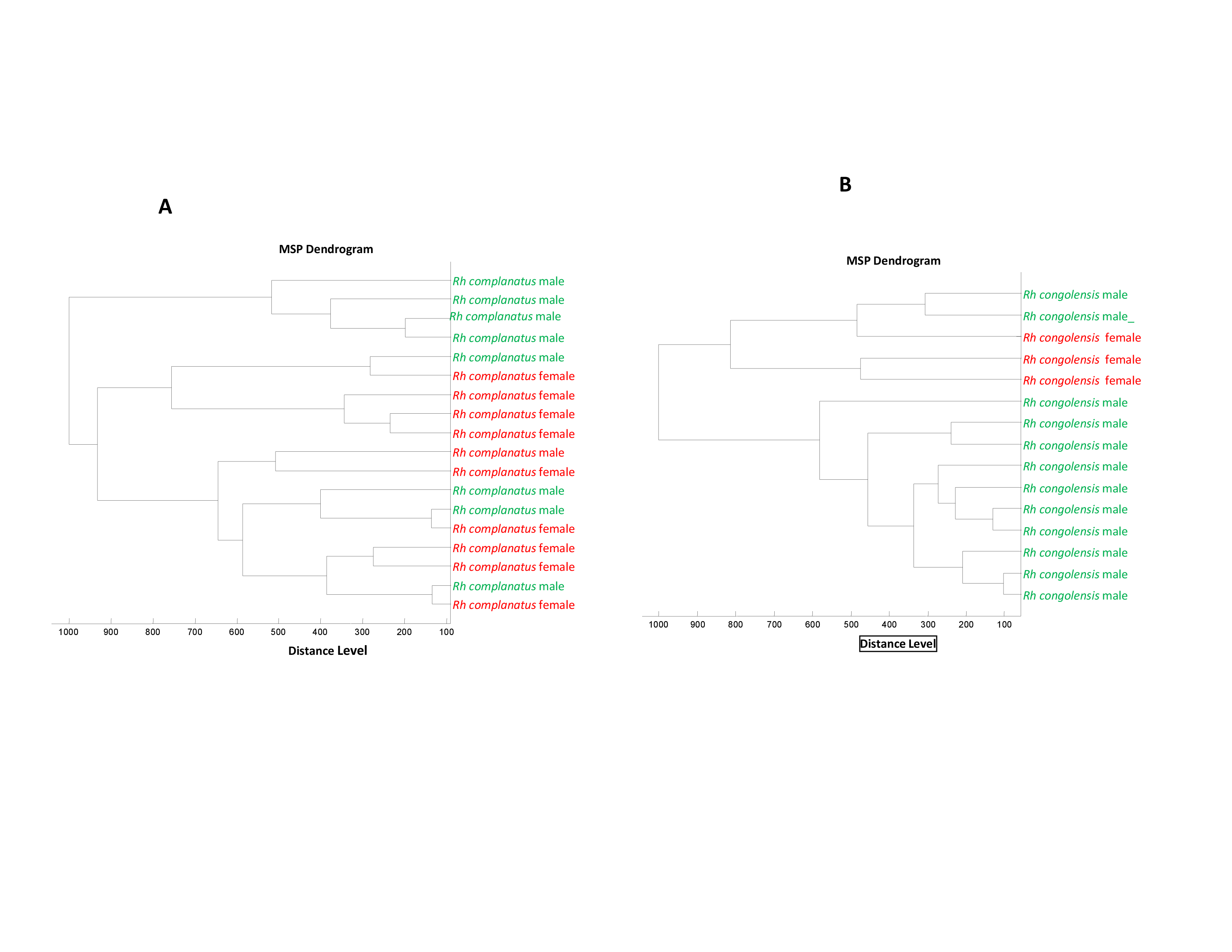

Supplement: Supplementary file 1 — Supplementary file1 (TIFF 688 KB) [file 10493_2021_629_MOESM1_ESM.tiff]
