# Supplementary material for: Using MALDI-TOF mass spectrometry to identify ticks collected on domestic and wild animals from the Democratic Republic of the Congo
Source: Exp Appl Acarol. 2021 Jun 19;84(3):637–57. doi: 10.1007/s10493-021-00629-z (PMC8257524; doi:10.1007/s10493-021-00629-z)
Supplement: Supplementary file 2 — Supplementary file2 (DOCX 32 KB) [file 10493_2021_629_MOESM2_ESM.docx]

Supplementary Table 1: list of different tick species and the GenBank accession numbers for the 16s, 12s rDNA and Cox1 mtDNA genes

| **Tick species (Morphological ID)** | **16S rDNA gene** | **12S rDNA gene** | **Cox1 mtDNA** **gene** |
| --- | --- | --- | --- |
| *Rhipicephalus complanatus* | KM894249- KM894253 | MT586096- MT586098 | MT646127- MT646131 |
| *Rhipicephalus congolensis* | KM894254- KM894257 | MT586099- MT586101 | MT646132- MT646133 |
| *Ixodes cumulatimpunctatus* | MN959769- MN959771 | MT586102 |  |
| *Rhipicephalus sp.* | MN959772- MN959774 | MT586103- MT586104 | MT646134- MT646136 |
| *Haemaphysalis muhsamae* | MN032115- MN032116 | MT586105 | MT646137 |
| *Amblyomma compressum* | MN032117- MN032119 | MT586106- MT586107 | MT646138- MT646139 |
| *Amblyomma exornatum* | MN032120- MN032122 | MT586108- MT586109 | MT646140- MT646142 |
